# Planar cell polarity: the Dachsous/Fat system contributes differently to the embryonic and larval stages of *Drosophila*

**DOI:** 10.1242/bio.017152

**Published:** 2016-03-02

**Authors:** Pedro Saavedra, Amy Brittle, Isabel M. Palacios, David Strutt, José Casal, Peter A. Lawrence

**Affiliations:** 1 Department of Zoology, University of Cambridge, Downing Street, Cambridge CB2 2EJ, UK; 2 Department of Biomedical Science, The University of Sheffield, Sheffield S10 2TN, UK

**Keywords:** Dachs, Dachsous, Fat, Epidermis, Planar cell polarity

## Abstract

The epidermal patterns of all three larval instars (L1–L3) of *Drosophila* are made by one unchanging set of cells. The seven rows of cuticular denticles of all larval stages are consistently planar polarised, some pointing forwards, others backwards. In L1 all the predenticles originate at the back of the cells but, in L2 and L3, they form at the front or the back of the cell depending on the polarity of the forthcoming denticles. We find that, to polarise all rows, the Dachsous/Fat system is differentially utilised; in L1 it is active in the placement of the actin-based predenticles but is not crucial for the final orientation of the cuticular denticles, in L2 and L3 it is needed for placement and polarity. We find Four-jointed to be strongly expressed in the tendon cells and show how this might explain the orientation of all seven rows. Unexpectedly, we find that L3 that lack Dachsous differ from larvae lacking Fat and we present evidence that this is due to differently mislocalised Dachs. We make some progress in understanding how Dachs contributes to phenotypes of wildtype and mutant larvae and adults.

## Abstract (precis)

**Summary:** *Drosophila* utilises the Ds/Ft system differently as it develops. Localised expression of *four-jointed* in the tendon cells may help polarise all rows of denticles in late larval stages.

## INTRODUCTION

During development, the larva of *Drosophila* undergoes three moult cycles and increases considerably in size. The cuticle of the first larval stage (L1) is formed by the embryonic epidermis; there are two subsequent stages (L2 and L3) and in each the epidermis secretes the cuticle of the next stage (Campos-Ortega and Hartenstein, 1997) Thus, the L1 makes the L2 cuticle and then moults to L2 which secretes the cuticle of L3 and the L3 secretes the pupal cuticle. The cuticles of all three larval stages are similarly patterned (Szabad et al., 1979; Dambly-Chaudière and Ghysen, 1986) leading to the reasonable assumption that the three stages are built by the same mechanisms; however, we showed that this is not the case (Saavedra et al., 2014). The larval cuticle shows a simple pattern and is suitable for genetic analysis (Bejsovec and Wieschaus, 1993; Alexandre et al., 1999; Wiellette and McGinnis, 1999). The ventral surface of each abdominal segment is decorated by about six or seven mediolateral rows of little cuticular hooks, or denticles (Lohs-Schardin et al., 1979; Martinez-Arias, 1993). Rows 1 and 4 point anteriorly, rows 2, 3, 5 and 6 posteriorly. The larval cells do not divide during growth, but instead increase in size by polytenisation (Pearson, 1974; Saavedra et al., 2014). It therefore seemed likely that the cells of L1 that made denticles of a particular row would also make denticles of that same row in L2 and L3 (Szabad et al., 1979; Dambly-Chaudière and Ghysen, 1986). We recently showed this expectation to be false; that actually the epidermal cells rearrange by convergent extension between the embryo and L2 and both the fates and polarities of individual cells change (Saavedra et al., 2014). It would appear therefore that the pattern of denticle rows is built afresh as L1 develops towards the L2.

What is known about the systems that build the patterns and polarities of the denticles in the three larval instars? Much work has been done on the L1 where the allocation of epidermal cells to rows is partially understood (Ingham and Martínez-Arias, 1992; St Johnston and Nusslein-Volhard, 1992; Hatini and DiNardo, 2001; Sanson, 2001). From its formation in the early embryo, each segment of the epidermis of the larva is divided into an anterior and a posterior compartment by cell lineage (Ingham and Martínez-Arias, 1992; St Johnston and Nusslein-Volhard, 1992). Ventrally, and in each segment, a stripe of Wingless is made by a single row of cells at the back of the anterior compartment (dependent on Hedghog, a protein emanating from the adjacent posterior compartment); Wingless is thought to spread anteriorly and posteriorly from the cells that make it. The resulting morphogen gradients are thought to pattern both compartments (Alexandre et al., 1999).

How the different rows acquire their polarity is not clear. In L1, the polarity may depend, directly or indirectly, on the slope of the Wingless and Hedgehog gradients; indeed if Wingless, or Hedgehog, are artificially expressed along the midline then the denticles turn 90° to point towards, or away from, the new source (Colosimo and Tolwinski, 2006). It is consistent with this model that at all larval stages, the denticle rows 2, 3, 5 and 6 of the anterior compartment point backwards, up the presumed gradients of Wingless. Also, the denticle rows 0 and 1 of the posterior compartment point forwards, again up the presumed gradient of Wingless (which is assumed to peak at its source, the most posterior cell row of each anterior compartment). However row 4 is not consistent with this simple model for its denticles point differently from the other rows of the anterior compartment, demanding a different explanation. Dilks and Dinardo discussed this and found a special explanation for both rows 1 and 4, which point anteriorly (Dilks and DiNardo, 2010).

**Fig. 1.**
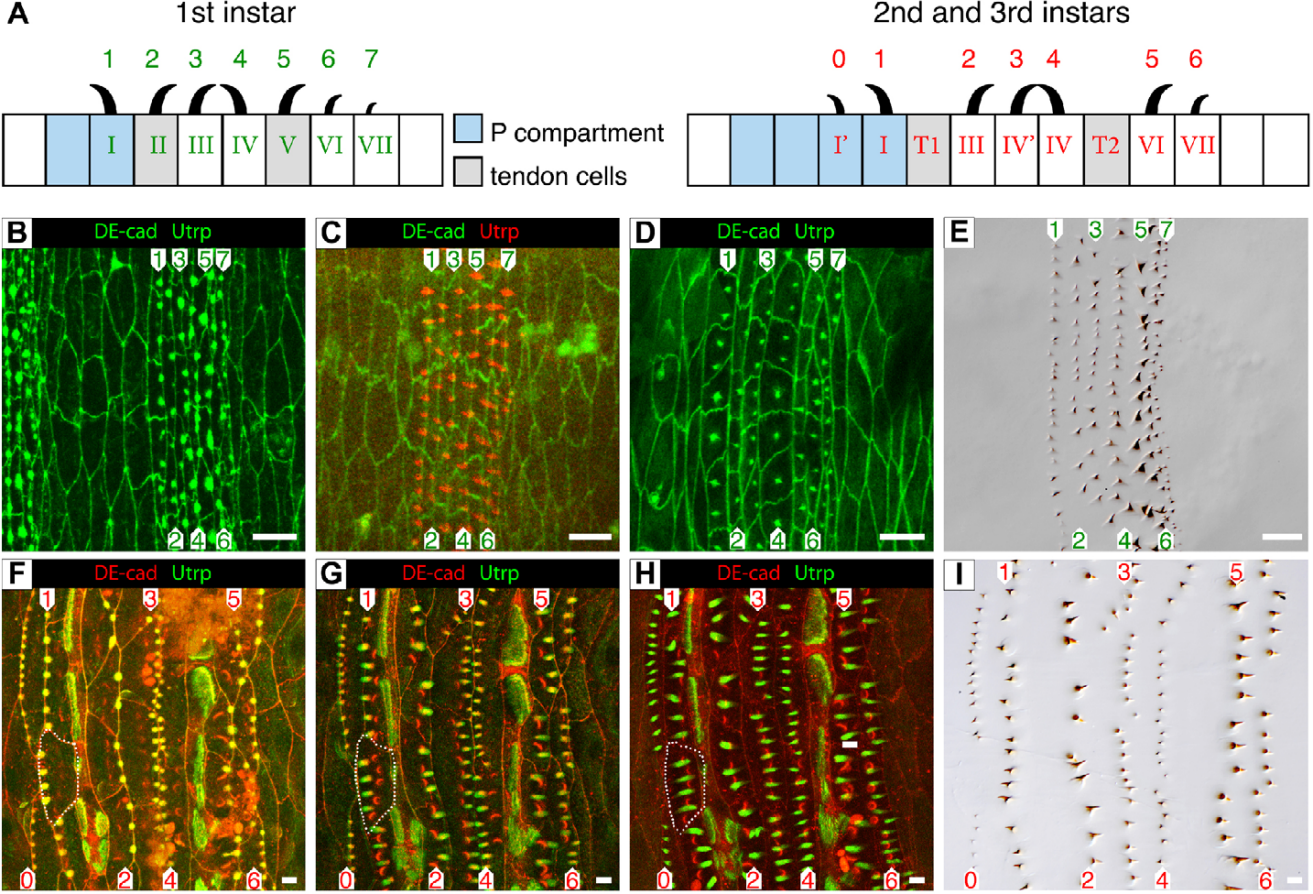
**Different modes of denticle development in embryo and larva.** Diagram (A, left) shows that in the wildtype embryo at L1 the seven rows of denticles are made by seven rows of cells, including two rows which are also tendon cells (Donoughe and DiNardo, 2011; Saavedra et al., 2014); a third row of tendon cells is found in the rear part of the anterior compartment (not shown). By contrast, in L2 and L3 the tendon cell rows do not produce denticles rows 2 and 5 denticles are now made by other cell rows. The development of the embryonic predenticles (B-D) shows the formation of predenticles (B) as largely unpolarised blobs which form at the posterior edges of the cells at stage 14. Then the predenticles become more defined and develop a backwards-facing polarity (C, stage 15). Then later the predenticles diminish and those of rows 1 and 4 move forwards to the middle of the cells (D, stage 16). By this time all denticles can just be detected (not shown) and they appear to point as they will in the definitive cuticle of L1 (E). Utrp (labelling actin structures) and DE-cad (labelling the apical cell membrane) are both shown in green in panels B and D; in C Utrp is red and DE-cad in green. (F-I) The development of predenticles followed *in vivo* in a single wildtype individual in pre-L3 is shown. A single cell is outlined in white which makes four predenticles that originate at its anterior margin (F), elongate (G) and move back to the middle of the cell (H). All predenticles form at the appropriate edge of the cell, at the front when they will form forward-pointing denticles and at the back when they will form backward-pointing denticles. Thus in (F) the predenticles of rows 3 and 4 are adjacent at the cell edge while the predenticles of rows 1 and 2 are far apart. The predenticles then move towards the cell centre (G) and come to be oriented (H) as the denticles that they later make (I). Compare rows 3 and 4 between F, G and H and note how these rows of predenticles move apart from each other. The diagrams in A show that, due to convergent extension, the rows of denticles are not made by the same cells in the three larval instars (L1, L2 and L3) (Saavedra et al., 2014). Utrp is green and DE-cad in red in panels F-H. Note in panel F-H, the confocal sections in the red channel show comma-like structures, which are due to autofluorescence emitted by the denticles of the L2 cuticle. Scale bars: 10 µm.

By contrast with the embryo and the L1, mechanisms that establish row polarity in L2 and L3 have been little studied. Note that three lines of *stripe* expression specify three separated rows of ‘tendon’ cells that attach to the muscles (Frommer et al., 1996; Hatini and DiNardo, 2001) – see Fig. 1A for a summary of the anatomy. Extensive remodelling occurs between L1 and L2; for example the tendon cells that make denticle rows 2 and 5 in L1 (Dilks and DiNardo, 2010) persist but do not make any denticles in L2 and L3 – these two denticle rows are made by other cells (Saavedra et al., 2014). Changes in the rows made by particular cells also mean that the polarities of individual cells alter between L1 and L2 (Saavedra et al., 2014).

In *Drosophila* (and elsewhere), planar polarity depends on at least two separate genetic systems, the Starry night (Stan) system and the Dachsous/Fat (Ds/Ft) systems (Casal et al., 2006; Lawrence et al., 2007; Goodrich and Strutt, 2011). The Ds/Ft system needs some introduction: the ability of Ds in one cell to bind to Ft in its neighbour depends on at least three factors; the level of Ds expression, the level of Ft expression and the activity of a regulator, Four-jointed (Fj) – Fj is a Golgi-resident kinase that phosphorylates both Ds and Ft, reducing the activity of the former while increasing the activity of the latter (Brittle et al., 2010; Simon et al., 2010). A collection of evidence argues that the adult hairs and bristles as well as larval denticles point to the neighbouring cell that has the most activity of Ds and away from the cell that has the most activity of Ft (Adler et al., 1998; Casal et al., 2002, 2006; Yang et al., 2002; Ma et al., 2003; Repiso et al., 2010; Donoughe and DiNardo, 2011; Saavedra et al., 2014; Rovira et al., 2015). Some evidence argues there is a gradient of Ds activity, with a peak at the back of the anterior compartment (Casal et al., 2002, 2006).

In this paper we describe the development of L3 epidermis and provide further evidence that the Ds/Ft system is the main instrument of polarity in the L2 and L3.

We report the following findings:

- In L1, the positions and orientations of the predenticles in the embryo for rows 1 and 4 form at the posterior of the cells even though the denticles will later point forwards (Dickinson and Thatcher, 1997; Price et al., 2006). However, in L2 and L3, for all the denticle rows, the edge of the cell where the predenticles form prefigures the orientation of the denticle, i.e. the predenticles for rows 0, 1 and 4 originate at the front of the cells and the predenticles for the other rows form at the back.
- We find differences in the action of the planar polarity genes when L1 is compared with L3. In both L1 and L3, removing the Stan system has very weak effects on the polarity of denticles. Removing the Ds/Ft system from embryos disturbs the placement of predenticles in L1 but has little effect on the final polarity of denticles (Lawlor et al., 2013; Marcinkevicius and Zallen, 2013). However, in L2/3, both the placement and polarity of the denticles depends on the Ds/Ft system.
- We now show evidence that Fj is produced strongly by the larval tendon cells and is predicted to reduce the activity of Ds and increase the activity of Ft therein, setting up a difference between the tendon cells and their neighbours that should direct the abutting denticle rows 4 and 1 to point forwards and the abutting rows 2 and 5 to point backwards, exactly as observed in the wildtype larva.
- We investigate how overexpressing Ds or Ft in a subset of cells influences the polarity of neighbouring cells and ask whether signal reception and propagation depends on the presence of Ft or Ds in the receiving cells. The results argue that the mechanisms of polarity signalling are different in the L3 and in the adult.
- To our surprise we found that *ds^−^* and *ft^−^* L3 larvae differ in phenotype. Our experiments suggest that this difference is due to interactions with the Dachs protein.

## RESULTS

### Description of predenticle and denticle development in the wildtype L1 and L3

In the embryo, the development of L1 predenticles and denticles has been well described (Dickinson and Thatcher, 1997; Moussian et al., 2006; Price et al., 2006). The predenticles develop into protrusions from the apical surface of the epidermal cell; they contain concentrations of filamentous actin and almost all of them develop into the cuticular denticles of L1. All the embryonic predenticles arise apically and at the posterior edges of the cells and all point posteriorly; this rule includes those predenticles of rows 1 and row 4, even though their cuticular denticles will later come to point anteriorly (Fig. 1) (Dickinson and Thatcher, 1997; Price et al., 2006). It was not clear when and how this change in polarity occurs. However, in late stage embryos, after the apical protrusion of predenticles, we found that only the cells producing rows 1 and 4 become more square and their predenticles move forwards from the back of the cells (Fig. 1D) (Saavedra et al., 2014). At this time, all the rows of predenticles, detected as actin staining, shrink and become blobs without any obvious polarity (Fig. 1D). In these individuals, at this stage, the nascent cuticular denticles can already be detected and those of rows 1 and 4 now point forwards (data not shown). The predenticles of rows 2, 3, 5 and 6 remain at the posterior edges of the cells and their denticles point posteriorly.

The embryonic cells persist from the embryo to the L3 without dying or dividing (Saavedra et al., 2014). Do the denticles of larvae, in the L2 and L3, develop as those in L1? During the latter parts of the L1, L2 and L3 stages, the cuticles of the next stages (L2, L3 and pupal cuticle, respectively) are secreted by the epidermis that underlies the previous cuticles. Following single individuals of L2 *in vivo* shows that each predenticle of L3 is first seen as a blob of actin that later forms into a spike. Each actin spike prefigures one cuticular denticle (compare Fig. 1F,G,H and I which are taken at different times in the development of a single individual). The predenticles of rows 0, 1 and 4 arise at the anterior interfaces of the cells and will point anteriorly. The remaining rows of predenticles (2, 3, 5 and 6) arise on the posterior margins of the cells and will point posteriorly (Fig. 1). Thus, unlike in the embryo, the positions of all predenticles presage the final orientations of the denticles. After the initial placements close to the posterior or anterior margins of the cells (Fig. 1F), all predenticles then relocalise to more central positions within the cells (Fig. 1H). The structure and behaviour of predenticles in L2 are similar to those in L3 (Fig. S1).

### Requirements for Ds/Ft system for predenticles and denticle development in L1 and L3

We have seen already in the larva that, even though the pattern of rows is conserved during growth and through the moult cycles, the identities of cells can change; thus, the same cell that contributes to row 4 in L1 may contribute to row 3 in L2 and L3 (Saavedra et al., 2014). This, and the differing ways the predenticles form in the embryo and larva, suggests that the mechanisms of PCP might also alter during development. To investigate we compared mutants in the Ds/Ft system with wildtype larvae.

The cells of the embryo undergo convergent extension between L1 and L2 (Saavedra et al., 2014) and we compared this process in wildtype, *ds^−^* and *ft^−^* larvae. The amount of convergent extension in mutant larvae is significantly reduced when compared to wildtype. In the wildtype the number of cells in the AP axis of a large ventral region of each segment (about 70 cells delimited by specific sensilla) increases from 14.3±0.2 in the embryo to 18.0±0.5 in L3 (Saavedra et al., 2014), however in the *ds* and *ft* mutants this increase is from about 14 (13.8±0.5 and 14.0±0.1, respectively) in the embryo to 17 in L3 (*n*=5, 16.4±0.4 and *n*=5, 17.1±0.5, respectively, *P*<0.001, Welch *t*-test). This small but real effect argues that cell rearrangement at convergent extension is not the same in mutants and wildtype, and therefore that the Ds/Ft system does contribute to this aspect of the wildtype phenotype. In both *ds* and *ft* mutants and in the wildtype the total number of epidermal cells in the measured region does not change during convergent extension (data not shown).

**Fig. 2.**
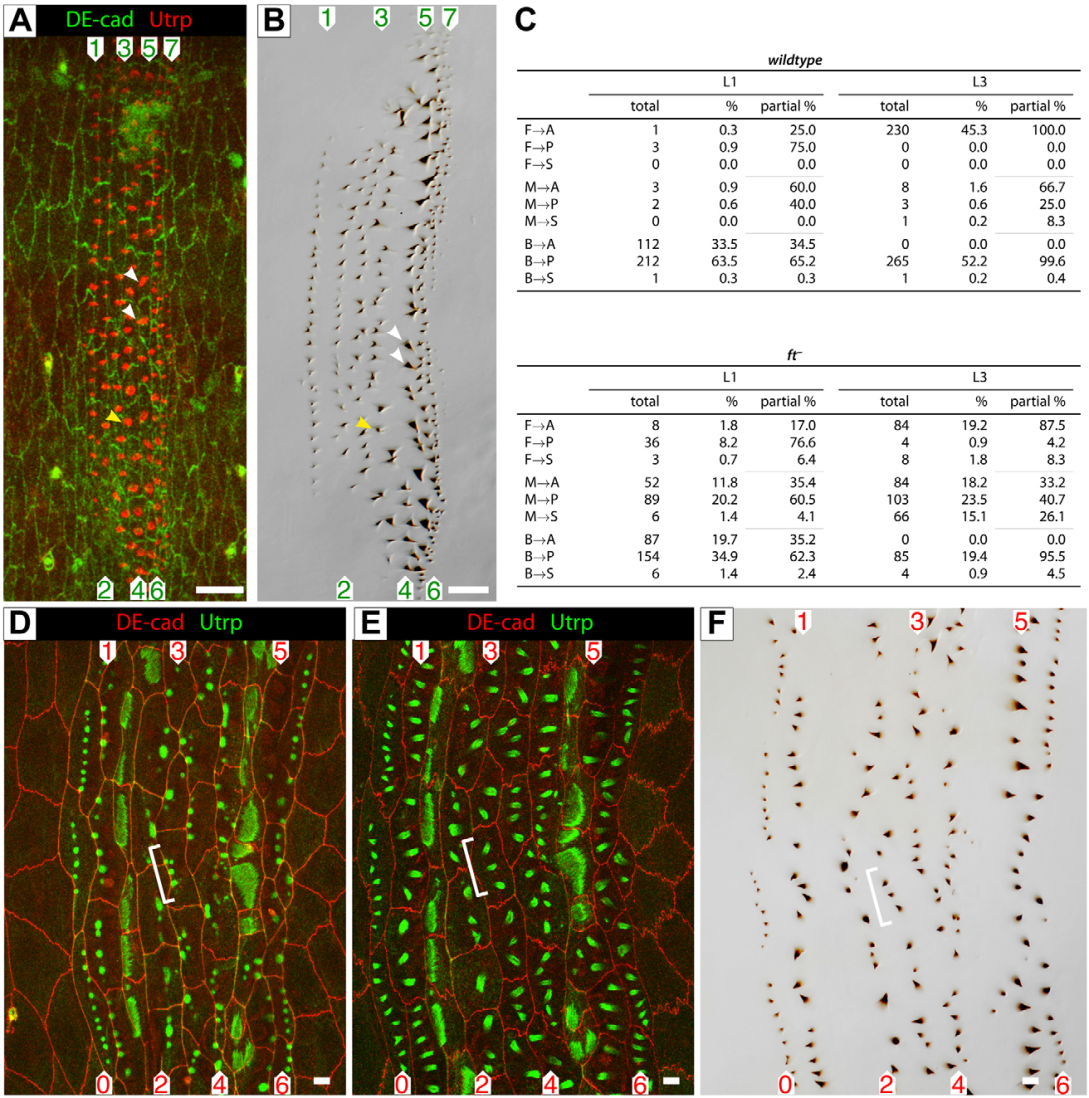
**Placement and development of predenticles; effect of *ft^−^.*** (A,B) In a single *ft^−^* embryo we show the placement of predenticles and the normal denticle orientation in the corresponding L1 cuticle. White arrowheads mark two predenticles that originate in the middle of the cell but give rise to backwards-pointing denticles. The yellow arrowhead points to a predenticle at the anterior of the cell, yet it forms a posteriorly oriented denticle. This and the normal orientation of denticles in B and C illustrates that, in *ft^−^*, even though placement is awry, mechanisms that orient denticles in the wild type L1 remain intact. Utrp is red and DE-cad in green in panel A. (C) Quantitation that shows how placement of the predenticle in the cell, front, middle or back correlates with the orientation of the corresponding denticle, pointing anteriorly, sideways or posteriorly, both in L1 and L3 and in wildtype and *ft^−^* larvae. (D-F) Three images from one individual showing the formation and maturation of the predenticles and the orientation of the denticles. Three particular predenticles and their corresponding denticles are marked. Utrp is green and DE-cad in red in panels D and E. Scale bars: 10 µm.

**Fig. 3.**
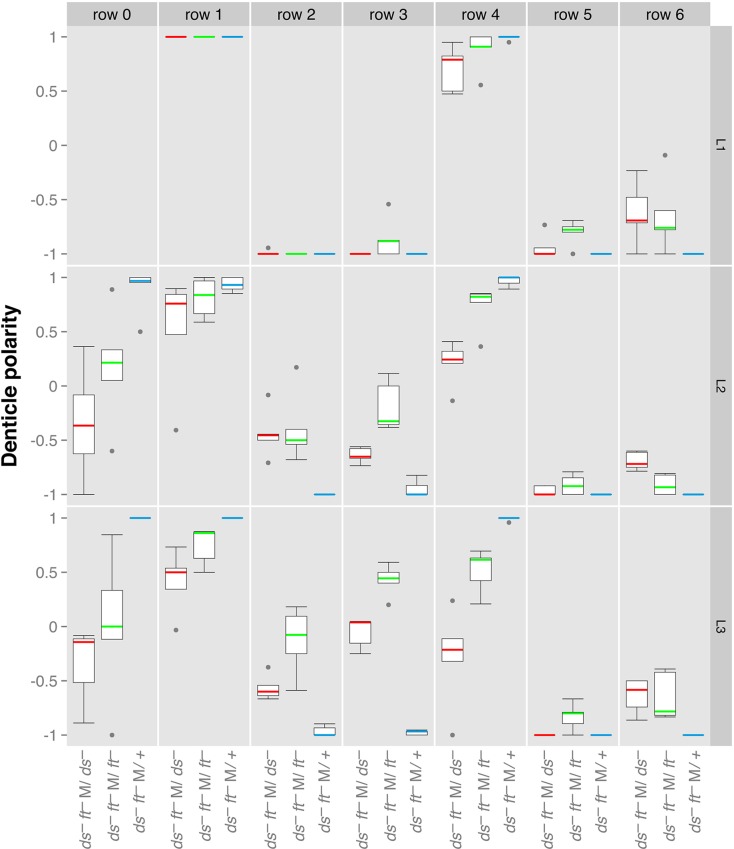
**Denticle orientation in wildtype, *ds^−^* and *ft^−^* larvae.** Orientations are scored by eye (double blinded, see Materials and Methods) and shown as boxplots (McGill et al., 1978). In L1 there is little effect of the mutants. In L3, *ds^−^* (red) and *ft^−^* (green) differ from each other, particularly in rows 2, 3 and 4. *ds^−^ ft^−^/+* (blue) resemble wildtype in phenotype. The wildtype genes were removed both maternally (M) and zygotically (Z). Denticle polarity ranges from −1, where all the denticles in a row are pointing posteriorly, to +1, where all the denticles are pointing anteriorly.

When *ds* and/or *ft* are missing from the embryo (the genes were removed both maternally and zygotically), the placements of predenticles in L1, especially of rows 3-5 are seriously disturbed (Donoughe and DiNardo, 2011) (Fig. 2). Nevertheless, in L1 the denticle polarity is little different from the wildtype (Donoughe and DiNardo, 2011; Marcinkevicius and Zallen, 2013) (Fig. 3), showing that any consequences of the misplacement of the predenticles are repaired by unknown mechanisms that build polarity subsequently. However, these unknown mechanisms do not operate in the later larval stages for, in L2 and L3, both in *ds^−^* and *ft^−^* (again, the genes were removed maternally and zygotically), several rows of denticles become disarrayed and their polarity is disturbed (Casal et al., 2006) (Fig. 3). But even in these mutants there is still a correlation between where the predenticles are placed in the cell membrane and the orientation of the corresponding denticles. In *ft^−^*, during the development of the L3 cuticle, almost all predenticles at the front edges of the cell make denticles that point anteriorly and almost all the predenticles placed at the back edge of a cell make denticles that point posteriorly. However predenticles that are formed away from the cell membranes (labelled M in Fig. 2C) give rise to denticles of various and unpredictable orientations. Thus, in L1 there are two mechanisms that contribute to the polarity of denticles, the first, depending on Ds/Ft system, places the predenticles and the second, which is independent of the Ds/Ft system, orients the final denticles. This second mechanism seems to be missing in L2 and L3; there the effects of loss of the Ds/Ft system on placement of the predenticles is sufficient to explain the denticular phenotypes.

### The distribution of *fj* expression

In wing imaginal discs, Ds protein exhibits planar polarity by accumulating unequally at the cellular interfaces, more at the distal face of the cells and less at the proximal face (Brittle et al., 2012). This distribution of Ds reflects the distribution of intercellular bridges; each one is a heterodimer consisting of Ds in the membrane of one cell and Ft in the membrane of its neighbour (Ma et al., 2003; Casal et al., 2006; Matakatsu and Blair, 2006). If Ds is overexpressed in a small clone of cells in the anterior compartment of an adult abdomen, the polarity of hairs and bristles in adjacent wildtype cells turns to point inwards, towards that clone, and this change in polarity spreads several cells away from the clone. If Ds is removed from a clone the surrounding hairs point away from the clone; both experiments suggesting that the orientation and distribution of Ds-Ft bridges that form between the abutting membranes of any two cells determines their polarities (Casal et al., 2002, 2006). As is consistent with the known action of Fj, clones of cells within which either *ft* or *fj* are overexpressed change the polarity of wildtype cells so that, in the adult, the bristles point away from the clone. Utilising these and other findings, we built a model of the L3 segment in which each cell or part of a cell makes a comparison between its neighbours and becomes polarised to point its denticles towards that neighbour cell that has the most activity of Ds (Repiso et al., 2010; Saavedra et al., 2014; Rovira et al., 2015).

**Fig. 4.**
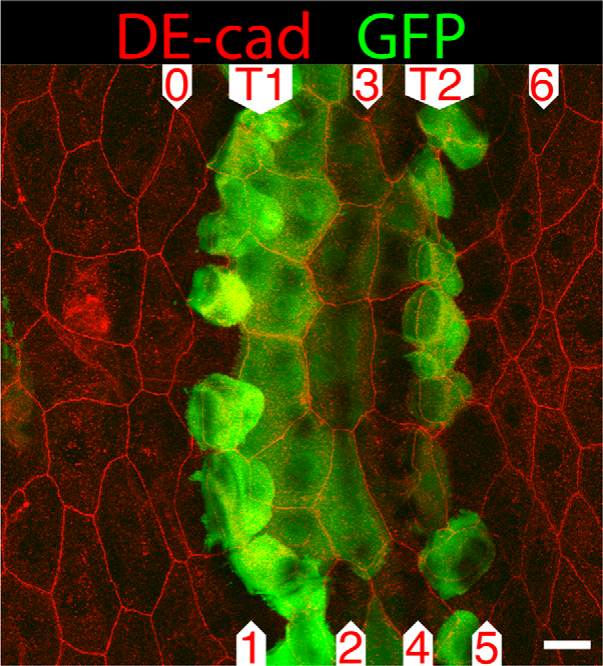
**Expression pattern of *four-jointed* in L3.** *fj.Gal4* drives *UAS.GFP* (green) in the ventral epidermis. There is strong expression in the tendon cells, T1 and T2 with moderate expression in the cells of row 2 and a trace in row 3. Compare Fig. 1A. DE-cad in red. Scale bar: 10 µm.

We mapped expression of *fj* in L3 and found it to be locally and strongly expressed in the rows of tendon cells T1 and T2 that lie between denticle rows 1 and 2, and between 4 and 5, respectively (Fig. 4) (Saavedra et al., 2014). Thus, Fj could help produce dips in the activity of Ds and/or peaks in the activity of Ft within the tendon cells and these fit with the wild type pattern in which all denticles point outwards from the nearest tendon cells (in which the Ds activity should be low). There is also some expression of *fj* in the three rows of cells between T1 and T2, most in row 2 and less in rows 3 and 4 (Fig. 4). Thus, if the Gal4 line we used faithfully reflects the expression of the endogenous *fj* gene, we can understand why row 3, cited midway between T1 and T2, points backwards (Rovira et al., 2015). However, the distribution of *fj* cannot be the only driver of the pattern of polarity as *fj*^–^ larvae have a near-normal phenotype (our own data, not shown, and Donoughe and DiNardo, 2011) arguing that other mechanisms also support the wildtype phenotype.

### Over-expressing genes in a specific subset of cells

#### Results in a wildtype background

The relevance of the Ds/Ft system to the different larval stages can also be assessed by over-expressing *ds* in a localised domain: in the adult wing and abdomen the polarity of nearby wildtype cells is altered so that they point towards this source of extra Ds (Casal et al., 2006). In L1, overexpressing *ds* in the posterior compartment affects placement of the predenticles in rows 2 and 3 (Lawlor et al., 2013) but has no corresponding effect on the polarity of the denticles, which are almost normal (Fig. S2). The same experiment in L2 and L3 gives, for rows 2 and 3, predenticles at the front of the cells and denticles that point in mixed directions instead of backwards as in the wildtype (Fig. S2) (Repiso et al., 2010; Donoughe and DiNardo, 2011).

**Fig. 5.**
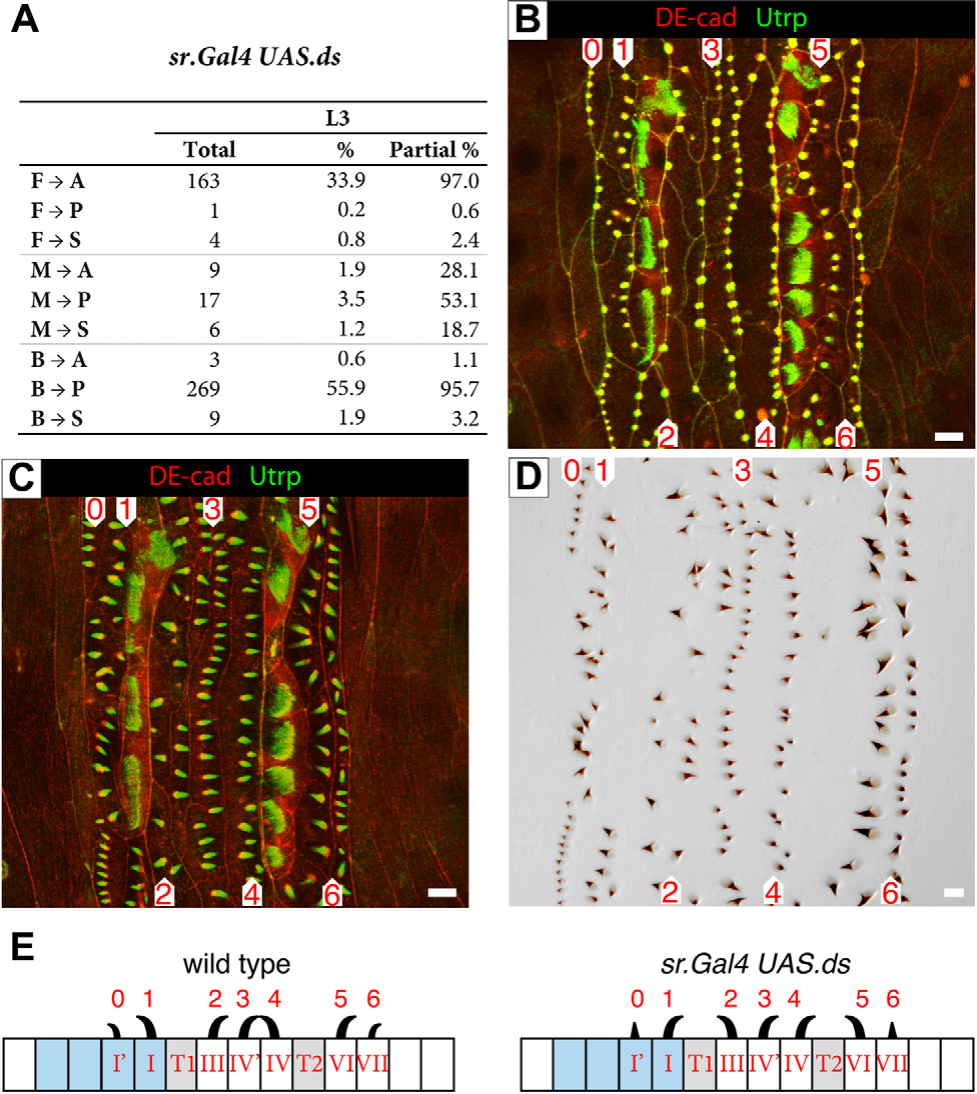
**The effects of overexpressing *ds* in the tendon cells.** (A) Quantitation of predenticle placement as in Fig. 2C. Effects on L1 are slight (not shown), but effects on L3 are substantial. The data in A shows the relationship between placement of predenticles and orientation of denticles in L3. In L3, predenticle rows 4 and 5 are mostly found at the opposite sides of the cell (than in wildtype) and corresponding denticle rows reversed, rows 1 and 2 are both disturbed. In L3 and as before there is a strong correlation between placement of the predenticle at either the front (F) or the back (B) of the cell with an anterior (A) or posterior (P) orientation of the denticle. When predenticles are found in the middle of the cell (M) then the orientation of the denticle is unpredictable and may even be sideways (S). (B-D) An individual imaged at two time points (B,C) and the denticle pattern (D). Compare with the wildtype in Fig. 1 and the row data for this genotype in Fig. 3. Utrp is green and DE-cad in red in panels B and C. (E) Diagram of rows and denticles in L3, style as in Fig. 1A. Note that in *sr.Gal4 UAS.ds*, denticle polarity of rows 1, 2, 4 and 5 is reversed.

In a new experiment, we over-expressed *ds* locally in the tendon cells (see below). In L1 there was little effect but in L3, both placement and the polarity of rows 1, 2 were strongly affected and rows 4 and 5 almost completely reversed (Fig. 5). Note that row 3 has normal polarity, even though it is equidistant from T1 and T2. These localised overexpression experiments confirm that the Ds/Ft system makes a smaller contribution to the final denticle polarity in the embryo than to the denticle polarity of the later larvae (Donoughe and DiNardo, 2011; Marcinkevicius and Zallen, 2013).

#### Overexpressing *ds* or *ft* locally in a background lacking Ds or Ft

In both the adult (Casal et al., 2002, 2006; Ma et al., 2003) and larva (Repiso et al., 2010; Rovira et al., 2015), cells overexpressing Ds change the polarity of neighbouring cells, changes that can propagate to the next neighbours and beyond. In the adult abdomen both Ds and Ft are required in those cells that respond to a polarising signal; for example a signal coming from a cell containing excessive Ds (the ‘sending cell’) changes the polarity of an abutting cell (the ‘receiving cell’) if that cell is wildtype but not if it lacks Ds (Casal et al., 2006). However, Donoughe and DiNardo (2011) found in the larva that *ds^−^* receiving cells can be repolarised by neighbouring cells that overexpress Ds. It follows that adult and larval epidermal cells differ. In addition, in the larva they detected propagation to *ds^−^* cells beyond the abutting receiving cells. Such a propagation effect, if it could be confirmed, would challenge any model that requires Ds/Ft heterodimers to be the only effective agent in intercellular communication by the Ds/Ft system. Such heterodimers could not form between two cells if both have Ft but neither have Ds.

**Fig. 6.**
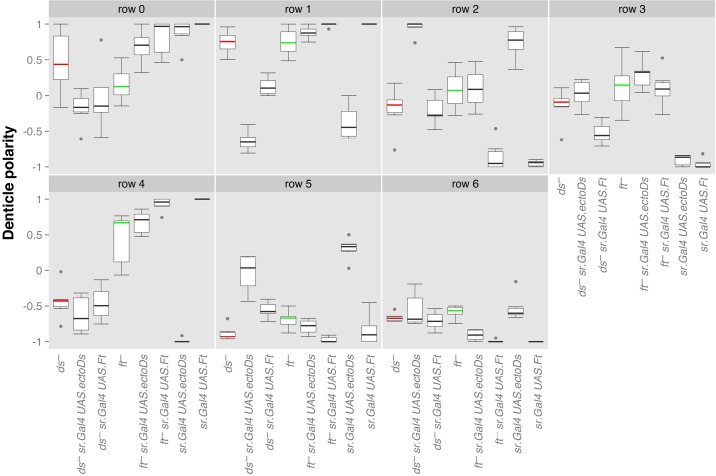
**Overexpression of *ds* and *ft* in the tendon cells of mutant L3 larvae.** Denticle orientations in L3 are shown for a number of genotypes listed below, in which we record the orientations of denticles per row. *ds^−^* is highlighted in red and *ft^−^* in green for clarity. Note that the last column, where *ft* is expressed in the tendon cells of a wildtype, produces a near-wildtype phenotype as expected – however, overexpressing *ds* in the tendon cells reverses row 4 and 5 and seriously disturbs row 1 and 2. Rows 0 and 6 are similarly disturbed in both the ‘experiments’ (*UAS.ectoDs* in *ds^−^*, *UAS.ft* in *ft^−^*) and the ‘controls’ (*UAS.ectoDs* in *ft^−^*, *UAS.ft* in *ds^−^*), see text.

To investigate further we overexpressed *ds* or *ft* in the rows of tendon cells of *ds^−^* or *ft^−^* larvae (Fig. 6). It is pertinent that Donoughe and DiNardo's picture of the anatomy of L2 and L3 was flawed: they, like us at that time, did not realise that these tendon cells (T1 in Fig. 1A) intervene between the cells of the posterior compartment that make row 1 and the responding anterior cells of row 2 (Donoughe and DiNardo, 2011). Also the tendon cells of T2 lie between rows 4 and 5 (Fig. 1A). Our images suggest that, apart from occasional places where the T1 cells are interrupted, there is no direct contact between the posterior compartment cells of row 1 and the row 2 cells, at least at the apical surface. We study four different rows of denticles (1, 2, 4 and 5) that adjoin the ectopic sources of Ds or Ft and two rows that are two cells away from these sources (0 and 6). We find that particular *ds^−^* rows are clearly repolarised by neighbouring cells overexpressing *ds* and that particular *ft^−^* rows are strongly repolarised by neighbouring cells overexpressing *ft.* The effects vary from row to row. Such effects on neighbouring mutant cells do not occur in the adult abdomen (Casal et al., 2006). Our results confirm and extend Donoughe and DiNardo's (2011) finding for the larva.

Even though it appears that there might be a weak ‘propagation’ to rows 0 and 6, we should consider the results of control experiments: first, *ds* was overexpressed in the tendon cells of *ft^−^* larvae and did not show any clear repolarisation of neighbouring cells (rows 1, 2, 4 and 5). This result would be expected because there should be no Ft in either sending or receiving cells and therefore no way that Ds−Ft heterodimers could form. However this control experiment still yields some changes in rows 0 and 6 and these cannot be due to propagation. Second, *ft* was overexpressed in the tendon cells in *ds^−^* larvae and there are some slight effects on several rows, but they are weak compared to the results with overexpression of *ds* in *ds^−^* (Fig. 6). Study of both the experiments proper and the controls suggest that the changes to polarity in rows 0 and 6 are independent from the polarity of their neighbours. We conclude therefore that in experimental larvae that lack Ds or Ft, there are strong effects on the polarity of the immediate neighbours of sending cells (Donoughe and DiNardo, 2011) but these changes are not propagated to next neighbours.

### Experiments in which single genes are removed, the function of Dachs

We expected that, because Ds and Ft function as heterodimers, removing either gene should produce the same phenotype. But this is not so: *ds^−^* and *ft^−^* larvae both have seriously disturbed polarities in all rows except for rows 5 and 6. However they differ strongly in the denticle polarities of rows 3 and 4 and less so in the orientation of row 2 (Figs 3 and 6). This difference is sufficiently clear to allow the sorting of *ds^−^* from *ft^−^* larvae by simple inspection in the microscope. We report below that this difference is related to the Dachs (D) protein. In the wildtype, Dachs protein is attracted to the cell membrane by Ds and repelled and/or degraded by Ft, particularly by means of its intracellular domain (Mao et al., 2006; Rogulja et al., 2008; Bosveld et al., 2012; Brittle et al., 2012). As a result, Dachs becomes localised to the region of the cell membrane that is rich in Ds protein and poor in Ft (Mao et al., 2006). In many current models this asymmetry of Dachs is seen to be crucial for PCP, for mechanical effects on cell shape (Mao et al., 2011; Bosveld et al., 2012) and/or the Hippo pathway (Rauskolb et al., 2011).

**Fig. 7.**
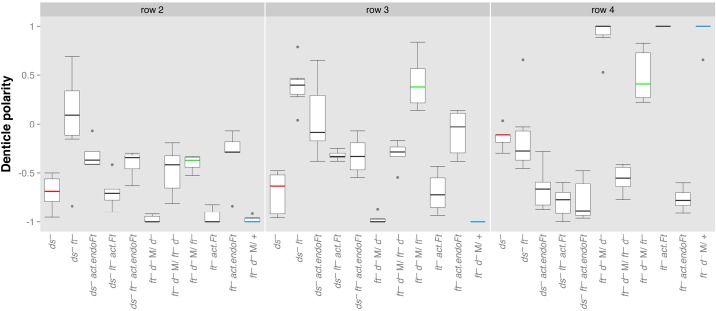
**The effects of removing Dachs and generalised overexpression of *endoFt* or *ft* on denticle orientation in L3.** The phenotype of *ds^−^* (shown in red) and *ft^−^* (green) are compared with controls, *ds^−^ ft^−^/+* (blue) and the effects on various genotypes of removing Dachs or ubiquitously overexpressing either *ft* (*act.ft*) or the intracellular portion of Ft (*act.endoFt*) reported. In L3, *ds^−^* (red) and *ft^−^* (green) differ from each other, particularly in rows 2, 3 and 4. *ft^−^ d^−^ M/+* (blue) resemble wildtype in phenotype. M signifies that the maternal expression of *ft* and *dachs* was removed. A Welch *t*-test comparison between *ds^−^* and *ft^−^* larvae shows that these two genotypes are different (*P*-values are 0.018, 0.000036, and 0.001 for rows 2, 3, and 4, respectively), whereas the addition of endoFt (*ds^−^ endoFt* and *ft^−^ endoFt* larvae) produces significantly similar phenotypes (*P*-values of 0.874, 0.497 and 0.359).

In L1 (Marcinkevicius and Zallen, 2013), in L3 (Fig. 7) and in the adult (Mao et al., 2006), *dachs* mutants are only slightly abnormal. However, in *ft^−^* and *ds^−^* adult wings and in the anterior compartment of the abdomen the phenotype includes whorly denticle polarity and misshapen organs; the immediate cause of this is thought to be mislocalised Dachs (Pan et al., 2013). The mislocalised Dachs would be expected to give abnormal output into PCP and into the Hippo pathway to induce this, an adventitious phenotype. Accordingly, removing Dachs from *ft^−^* adults changes the mutant phenotype towards the wildtype (Cho and Irvine, 2004). Adding endoFt to *ft^−^* adults has a similar effect, also ‘rescuing’ this adventitious phenotype (Matakatsu and Blair, 2006). Indeed when cells are flooded with endoFt, Dachs departs from the cell membrane whether the cells are cells are *ft^+^* (Rodrigues-Campos and Thompson, 2014) or *ft^−^* (Fig. S3).

**Fig. 8.**
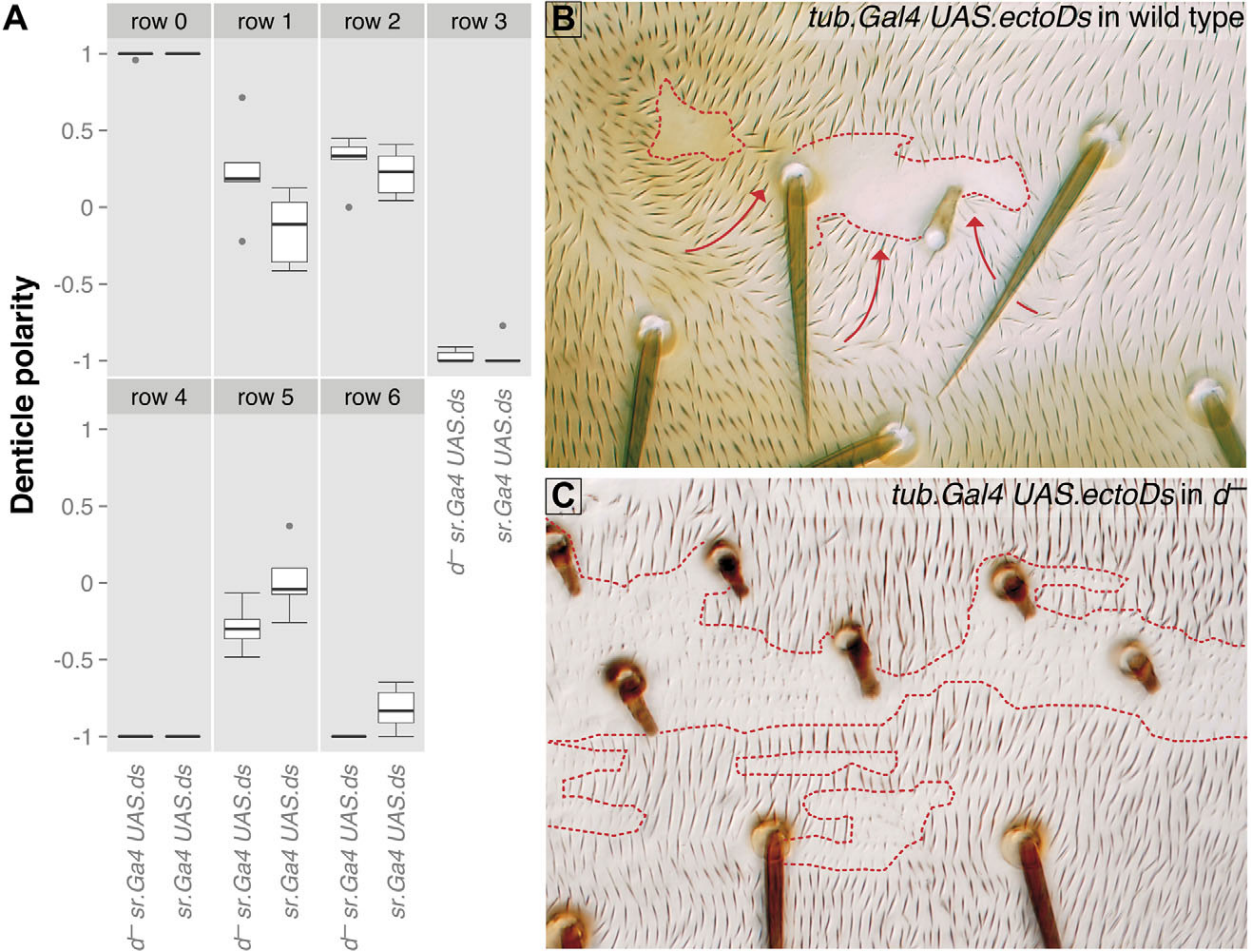
**Overexpression of *ds* in larvae and adults lacking Dachs.** (A) In the larva, loss of Dachs does not affect the response of neighbouring cells to overexpression of *ds* in the tendon cells of L3. (B,C) In the adult, loss of Dachs blocks the response of receiving cells to overexpression of *ds* in adjacent sending cells. In B the clone of expressing cells is marked with *pawn* and *shavenoid*, in C with *pawn* alone. Repolarisation behind the clone in *d^+^* adults is obvious in B and lacking in *d*^–^ (C). Red dotted lines in B and C define the contours of the clones, red arrows in B indicate areas around the clone with reversed polarity.

Attempting to understand the function of Dachs further, we overexpressed Ds in the tendon cells, but in a *dachs* mutant background. We found that denticles of rows 1, 2, 4 and 5 have their orientations largely reversed, exactly as in a *dachs^+^* background (Fig. 8A). This shows that Dachs is not essential in the larval receiving cells for them to respond to this PCP signalling. However, new findings in the adult lead to the opposite conclusion: overexpressing *ds* at levels that would normally change the polarity of several rows of surrounding wildtype cells (Casal et al., 2006), do not do so if these receiving cells lack the Dachs protein (Fig. 8B,C). Again there appears to be a difference between the requirement for Dachs in larva and adult.

## DISCUSSION

In the three larval stages of *Drosophila* the pattern and polarity of cuticular denticles remains mostly the same. Nevertheless, the cells rearrange considerably and their identity changes (Saavedra et al., 2014). The mechanisms responsible for denticle polarity in L1 also differ substantially from those in L2 and L3; one can conclude that the pattern is not replicated as L1 metamorphoses into L2, but is substantially rebuilt. Consider row 1 and 4 which point forwards as an example. In the embryo, the orientation of rows 1 and 4 is partly dependent on the *stripe* and *short stop* genes (Dilks and DiNardo, 2010). But in the later stages we show that row 4 as well as the other rows is oriented largely by the Ds/Ft system, a system that has a detectable but a lesser role in L1.

We have proposed that, in the making of denticle rows of L3, several elements combine to produce a rolling landscape of Ds activity whose slopes are read out locally to polarise each of the 6-7 rows (Rovira et al., 2015). The results in this paper are consistent with this model, so for example if Ds is overexpressed in the tendon cells (where Ds activity is presumed to be low due to the expression of *fj* in those cells, Fig. 4) the orientation of all rows is altered, as expected from the model. However direct evidence for the model is still lacking as we have not been able to map the distribution of Ds or Ft in the larva. There are most likely other systems that contribute to the wildtype pattern, one possible contributor being the Wg protein.

### Cell biology of denticle development

Development of predenticles in larval stages was observed *in vivo*. In the embryo, the predenticles of all rows originate at the back of the cells and point clearly backwards; later those of rows 1 and 4 move towards the centre of the cell and then help form denticles that point anteriorwards. The predenticles of the other rows appear to remain at the back of the cells and then form denticles that point backwards. By contrast, in L2 and L3 the predenticles of rows 0, 1 and 4 originate at the front of the cells while the other rows form at the back. Our interpretation is again that the mechanisms of PCP change; in the early embryo all the cells have the same polarity and the predenticles point backwards, an outcome that depends on the Ds/Ft system. However, later in embryogenesis, polarity of the denticles is reset by different mechanisms that do not depend on Ds and Ft and involve all the rows, including 1 and 4 (which point forwards). These mechanisms can correct defects due to the loss of Ds or Ft. But later, in L2 and L3, both placement and polarity of the rows are organised afresh by the Ds/Ft system.

Independence between placement and orientation is also found in the *Drosophila* wing. There are some indications that prehairs in the wildtype wing may also shift during maturation. The prehairs initiate and elongate at the distal edge of the wing cells, but after extensive cell shape changes the mature hairs are found in the middle (Mitchell et al., 1983, 1990; Wong and Adler, 1993). In *fz^−^* wings the pre-hairs are formed in the centre of the cell yet the polarities of the cuticular hairs are still coordinated from cell to cell to make whorly patterns (Gubb and Garcia-Bellido, 1982; Wong and Adler, 1993). Similarly in Celsr mutant mouse hair follicles, the follicle itself, unlike in the wildtype, is unpolarised but the hairs the follicles form are oriented and make large-scale whorly patterns (Devenport and Fuchs, 2008). In the vertebrate ear there is also late-stage refinement of orientation in the wildtype, and in Vangl2 mice the late changes in polarity can include complete reversal to a near-wildtype phenotype (Copley et al., 2013). These observations show again that the acquisition of polarity is a complex multistep process and is not under the control of a single and direct pathway.

### Differences between the Ds/Ft system in larva and adult

There appear to be differences between how the Ds/Ft system functions in larva and adult. In the adult abdomen, ‘signalling’ cells, which have a higher or lower amount of Ds or Ft can change the polarity of neighbouring ‘receiving’ cells, but only if those have both the *ds* and *ft* genes (Casal et al., 2006). In 2006 this result surprised us because a clone overexpressing Ds should, even in a *ds^−^* receiving cell, pull Ft to the abutting membrane, causing asymmetry in the distribution of Ft within that cell. Such an asymmetry would be expected to polarise the receiving cell. Here we have confirmed and extended Donoughe and Dinardo's finding in the larva that *ds^−^* receiving cells are polarised by neighbouring signalling cells that overexpress Ds (Donoughe and DiNardo, 2011). We have shown the same result when the receiving cells are *dachs^−^*. Results that leave the situation in the adult abdomen unexplained. One possibility is that the adult cells can ‘receive’ the signal (i.e. that Ft might be pulled over to the membrane abutting the signalling cell) but they cannot ‘respond’ to the signal without Ds, Ft and Dachs being present in the cell.

### The role of Dachs

How much does Dachs contribute to the polarities of the denticle rows of the wildtype L3 and does it also contribute to polarities of mutant larvae? When Dachs is removed from wildtype larvae there is little change of the pattern suggesting its contribution to the wildtype may not be great. However, when Dachs is removed from *ft^−^* larvae the mutant phenotype alters considerably (Fig. 7). Mao et al. (2006) found a large increase in amounts of Dachs at the membrane, uniformly distributed, in the case of *ft^−^*, and a smaller amount in the case of *ds^−^*, also uniformly distributed. Probably therefore this mislocalised Dachs is at least partly responsible for both the *ft^−^* and *ds^−^* phenotypes, and particularly for the difference between them. Dachs is displaced from the membrane by endoFt (Rodrigues-Campos and Thompson, 2014) (Fig. S3) and thus when endoFt is added to *ft^−^* or to *ds^−^* larvae the phenotypes come to resemble *ft^−^ dachs^−^* larvae; indeed *ft^−^ act.endoFt* and *ds^−^ act.endoFt* are indistinguishable (Fig. 7). A result supporting our view that Ft and Ds make functionally equivalent contributions to PCP (Casal et al., 2006) and not differing contributions with Ft acting as a receptor and Ds as its ligand, as has often been averred (Reddy and Irvine, 2008).

Removing Dachs, or adding endoFt to *ds^−^* or *ft^−^*, produces larvae with similar denticle polarities; however we do not consider that these larval phenotypes have been ‘rescued’. Even in the adult, although the mutant phenotypes of *ds^−^* or *ft^−^* (misshapen wings and legs, resembling the phenotype of mutants in the hippo pathway) are partially repaired when Dachs is removed or endoFt is added there are still abnormalities in polarity: the hairs of the posterior compartment of the adult abdomen that point forwards in the *ft^−^* abdomen continue to point forwards in *ft^−^ dachs^−^* flies.

Earlier (Lawrence and Casal, 2013), we suggested that the Ds/Ft system has two separate functions: one mediated by the extracellular domains that form the intercellular bridges, and another localising Dachs at one side of each cell. In the absence of either Ds or Fat, the bridges cannot form, yet nevertheless there is mislocalisation of Dachs (Mao et al., 2006), which may cause a neomorphic phenotype – such as the whorly polarity in the abdomen of *ft^−^* adults. This perspective undermines the logic behind structure-functional analyses of the Ft molecule that depend on localising the part of the molecule responsible for an apparent ‘rescue’ in the adult (Matakatsu and Blair, 2012; Pan et al., 2013; Zhao et al., 2013).

### Genetic pathways and PCP

Geneticists investigating a developmental process start by identifying one or more genes whose loss causes the biggest phenotypic deficit. These genes can then define a ‘key pathway’. But in complex processes there are always other genetic systems than the main one; these other systems back up failures in the key pathway and help to ‘canalise’ the wild type phenotype (Waddington, 1942; Siegal and Bergman, 2002). Here, we have used a single model based on the Ds/Ft system in which the distribution of Fj and Ds can explain the orientation of all rows. However our results raise some difficulties with this simple view. For example if the output from the interaction of Ds with Ft were to go entirely through Dachs, then the phenotypes of *ft^−^* and *ft^−^ dachs^−^* larvae should be the same. But they are not, and this difference suggests that the Ft/Ds system has other *dachs*-independent outputs. It is not clear how significant these other outputs are. Developmental geneticists tend to underestimate the importance of alternative systems that support the wildtype phenotype, particularly as the contribution of each may only be visible in a background in which a key pathway is already broken. Selection can play games with these different systems, so as development proceeds, the predominant system at one stage may become a back up system at a later stage and vice versa. This is true of the Ds/Ft system that makes a minor contribution to the polarity of denticles of the L1 but a major contribution to L2 and L3.

### Polymorphism, evolution and developmental genetics

“… the organs of the larva are as highly differentiated and as specialised in their own way as those of the adults” (Wigglesworth, 1954)

Sixty years ago and without the benefit of genetics, Wigglesworth reflected on insect metamorphosis. He asked: how could such different forms as a maggot and a fly be built by one set of information? Similarly, how can one explain differing anatomies within one stage, such as castes in termites? Was this problem not related to another: how does a single set of information build, say, the first and third legs which have such different forms and functions? In trying to answer these questions we should seek to find the ‘internal description’ – how the animal defines and builds itself (Brenner, 2012), and distinguish that from the external description – how we describe the animal. Most describe Diptera as having only three ‘life forms’, larva, pupa and adult. But our earlier results (Saavedra et al., 2014) showed that the epidermal cells of L1 are radically remodelled and repatterned as they build L2 and L3. This argues that L2 and L3 constitute a different ‘life form’ from L1 because they are made in different ways. Consider the seven rows of denticles that are (presumably) functionally important in all larval stages and so it would seem that the final pattern (the external description) has been conserved by selection, even though the mechanisms used to build the seven rows (the internal description) have varied. Here we have shown that the way the Ds/Ft system is deployed also varies between L1, L2/3 and the adult.

## MATERIALS AND METHODS

### Mutations and transgenes

The FlyBase (dos Santos et al., 2015) entries for the genes and transgenes are the following: *DE-cad::GFP*: *shg*^*Ubi-p63E.T:Avic\GFP-rs*^. *DE-cad::tomato: shg*^*KI.T:Disc\RFP-tdTomato*^. *Dfd.YFP: P{Dfd-GMR-nvYFP}*. *fj.Gal4: P{GawB}fj*^*VG1*^. *ovoD1: P{w[+mC]=ovoD1-18}2La P{w[+mC]=ovoD1-18}2Lb. sqh.utrp::GFP: Hsap\UTRN*^Scer\UAS.P\T.T:Avic\GFP-EGFP^. *sr.Gal4: sr*^*md710*^. *tub.Gal4: P{tubP-GAL4}. tub.Gal80: P{tubP-GAL80}. UAS.ds: P{UAS-ds.T}. UAS.ectoDs: P{UAS-ds.ecto}. UAS.ft: P{UAS-ft.M}. UAS-Syn21.GFP: Avic\GFP*^*IVS.Syn21.10xScer\UAS*^. *act.ft: AttB{w+ ActP-FRT-polyA-FRT-ft–EGFP}* (Hale et al., 2015). *act.endoFt*: The CD2 signal peptide followed by 3×Myc tags was fused to FtΔ1-4410 using a *Sal*I site and cloned into attB-ActP-FRT-polyA-FRT for generation of transgenic line *AttB{w^+^ ActP-FRT-polyA-FRT-ΔECDft^−^EGFP}*.

### Experimental genotypes

Fig. 1. Panels B and D: *w; sqh.utrp::GFP/DE-cad::GFP*. Panels C, and E-I: *w; sqh.utrp::GFP/DE-cad::tomato*.

Fig. 2. Panels A-B, and D-F: *w; ds^UA071^ ft^15^ DE-cad::tomato/ft^G-rv^; sqh.utrp::GFP/+*.

Fig. 3. Females of genotype *y w hs.FLP/w; ds^UA071^ ft^15^ FRT40A/ovoD1 FRT40A* (ds^−^ ft^−^ M) were heat shocked in L3 and crossed to males of various genotypes to produce the following experimental genotypes: **ds^−^ ft^−^ M/ds^−^**: *w; ds^UA071^ ft^15^ FRT40A/ds^UA071^*; **ds^−^ ft^−^ M/ft^−^**: *w; ds^UA071^ ft^15^ FRT40A/ft^G-rv^*; **ds^−^ ft^−^ M/+**: *w/+; ds^UA071^ ft^15^ FRT40A/+*.

Fig. 4. *w; fj.Gal4/+; UAS-Syn21.GFP/+.*

Fig. 5. *w; sqh.utrp::gfp/DE-cad::tomato; sr.Gal4/UAS.ds*.

Fig. 6. **ds^−^**: *w; ds^UA071^ ft^15^ DE-cad::tomato/ds^UA071^ tub.gal80 FRT40A; sr.Gal4/+*. **ft^−^**: *w; ds^UA071^ ft^15^ DE-cad::tomato/ft^G-rv^; sr.Gal4/+*. **sr.Gal4 ectoDs**: *w; ds^UA071^ ft^15^ DE-cad::tomato DE-cad::tomato/CyO, Dfd.YFP; sr.Gal4/UAS.ectoDs*. **sr.Gal4 UAS.ft**: *w; ds^UA071^ ft^15^ DE-cad::tomato/CyO, Dfd.YFP; sr.Gal4/UAS.ft*. **ds^−^ sr.Gal4 ectoDs**: *w; ds^UA071^ ft^15^ DE-cad::tomato/ds^UA071^ ck FRT40A; sr.Gal4/UAS.ectoDs*. **ds^−^ sr.Gal4 UAS.ft**: *w; ds^UA071^ ft^15^ DE-cad::tomato/ds^UA071^ fj^d1^; sr.Gal4/UAS.ft*. **ft^−^ sr.Gal4 UAS. ectoDs**: *w; ds^UA071^ ft^15^ DE-cad::tomato/ft^G-rv^; sr.Gal4/UAS.ectoDs*. **ft^−^ sr.Gal4 UAS.ft**: *w; ds^UA071^ ft^15^ DE-cad::tomato/ft^G-rv^; sr.Gal4/ UAS.ft*.

Fig. 7. **ds^−^**: *w; ds^UA071^ fj^d1^/ds^38k^*. **ft^−^**: *w; ft^8^ fj^d1^ FRT40A/ds^UA071^ ft^G-rv^ fj^d1^; TM2/+*. **ds^−^ ft^−^**: *ds^38k^ ft^8^ fj^d1^/ds^UA071^ ft^15^ DE-cad::tomato; TM2/+*. **ds^−^ ft^−^ act.endoFt**: *w; ds^UA071^ ft^15^ FRT40A/ds^38k^ ft^8^ fj^d1^; act.endoFt/+*. **ds^−^ act.endoFt**: *w/y w hs.FLP122; ds^UA071^ pk^pk-sple-13^/ds^38k^ ft^8^ fj^d1^; act.endoFt/+*. **ft^−^ act.endoFt**: *w; ft^G-rv^ FRT40A/ds^38k^ ft^8^ fj^d1^; act.endoFt/+*. **ds^−^ ft^−^ act.Ft**: *w; ds^UA071^ ft^G-rv^ DE-cad::tomato/ds^UA071^ ft^G-rv^ fj^d1^; act.Ft/+*. **ft^−^ act.Ft**: *w; ft^8^ d^1^ FRT40A/ds^UA071^ ft^G-rv^ fj^d1^; act.Ft/+*. Females of genotype *y w hs.FLP/w; ft^G-rv^ d^GC13^ FRT40A/ovoD1 FRT40A* (ft^−^ d^−^ M) were heat shocked during L3, crossed to males of various genotypes to produce the following experimental genotypes: **ft^−^ d^−^ M/d^−^**: *w; ft^G-rv^ d^GC13^ FRT40A/d^GC13^*; **ft^−^ d^−^ M/ft^−^**: *w; ft^G-rv^ d^GC13^ FRT40A/ft^8^ FRT40A; Tub.Gal4/+*; **ft^−^ d^−^ M/ft^−^ d^−^**: *w; ft^G-rv^ d^GC13^ FRT40A/ft^8^ d^GC13^*; **ft^−^ d^−^ M/+**: *w/+ ; ft^G-rv^ d^GC13^ FRT40A/+*.

Fig. 8. Panel A: **d^−^ sr.Gal4 UAS.ds**: *w; d^GC13^ sqh.utrp::GFP/d^GC13^ pr cn; sr.Gal4/UAS.ds*; **sr.Gal4 UAS.ds**: *w; sqh.utrp::GFP/DE-cad::tomato; sr.Gal4/UAS.ds*. Panel B: **tub.Gal4 UAS.ectoDs clones in wild type**: *y w FL; FRT42D pwn sha/FRT42D tub.Gal80; UAS.ectoDs/tub.Gal4*. Panel C: **tub.Gal4 UAS.ectoDs clones in d^−^**: *y w hs.FLP/y w hs.FLP tub.Gal4 UAS.nls-GFP; d^GC13^ FRT42D pwn/d^GC13^ FRT42D tub.Gal80; UAS.ectoDs/+*.

### Immunostaining

Third instar wing disks were fixed in 4% formaldehyde and washed in PBS 0.1% Triton-X-100, and incubated with rat antibodies against D and Ft (Brittle et al., 2012) and a mouse monoclonal anti-Armadillo (Developmental Studies Hybridoma Bank, University of Iowa). Secondary antibodies used were anti-Rb Cy2, anti-mouse Cy5 (Jackson ImmunoResearch), and anti-Rat A568 (Molecular Probes).

### Microscopy

Observation of live embryos and larvae was carried out as in Saavedra et al. (2014) using a Leica inverted SP5 confocal microscope. Larval cuticles were prepared following Wieschaus and Nusslein-Volhard (1986), L3 larvae were sliced longitudinally before mounting and examined under a Zeiss Axiophot equipped with a Nikon D-300 camera.

### Quantification of cells in embryo and larva

For this we followed Saavedra et al. (2014).

### Scoring of denticles

These were scored blind using a mix of slides of genotypes that were unknown to the first observer. The first observer read out the denticles as pointing anterior, posterior or sideways and a second observer recorded this. This relatively quick process means that the observer can go along each row without taking his eyes from the microscope (experience showed that the use of buttons by this single observer was prone to error). For simplicity, the denticles classified as ‘sideways’ were divided equally into the anterior and posterior classes. For statistical analysis we used the R programming language and software environment (R Core Team, 2015). For all the plots at least 5 individuals were scored, each for a fourth or fifth abdominal segment; ‘denticle polarity’ was recorded as percentages. The scale ranges from −1 (all denticles pointing posteriorly) to +1 (all denticles pointing anteriorly), while 0 corresponds to no bias. Judging row identity, even in wildtype and more in mutants, can be tricky. For example, Figs. 2D and 2F show that, centrally, there are two denticulated cells between rows 2 and 4, instead of just one (see also Rovira et al., 2015). This problem must affect all of us who quantitate denticle orientation row by row.
